# Insight into the Role of Sulfur Confined within the Porous Carbon Structure in 2‑Chloroethyl Ethyl Sulfide Adsorption

**DOI:** 10.1021/acs.langmuir.6c05969

**Published:** 2026-10-27

**Authors:** Paola S. Pauletto, Marc Florent, Jingwen Wang, Piotr Borowski, Teresa J. Bandosz

**Affiliations:** † Department of Chemistry and Biochemistry, 14770 The City College of New York, 160 Convent Avenue, New York, New York 10031, United States; ‡ Faculty of Chemistry, Maria Curie-Sklodowska University, Pl. Marii Curie-Sklodowskiej 5, 20-031 Lublin, Poland

## Abstract

The interactions of 2-chloroethyl ethyl sulfide (CEES), a mustard gas surrogate, with porous carbons, whose surface features were tuned by sulfur insertion, were investigated in this study. The modification process introduced 0%, 5%, 10%, and 50% sulfur relative to the total pore volume of each carbon. Both surface chemistry and porosity were affected, and the extent of changes depended on the initial carbon surface features. CEES adsorption experiments were performed in a closed system, enabling simultaneous analysis of the surrogate adsorbed on the carbon surface and its vapors released into the headspace. The results showed that a gradual decrease in the porosity by sulfur insertion markedly hindered CEES adsorption. The measurement of the heat released upon complete filling of the pores revealed its dependence on accessible pores with the highest adsorption potentials. The analysis of the efficiency of CEES extraction from the pore system combined with DFT calculations suggested that at a low sulfur loading (5% of pore volume), confined sulfur promoted the retention of CEES molecules due to S_surface_–S_CEES_ dispersive interactions, emphasizing the importance of deposited sulfur for separation and extraction processes.

## Graphical abstract

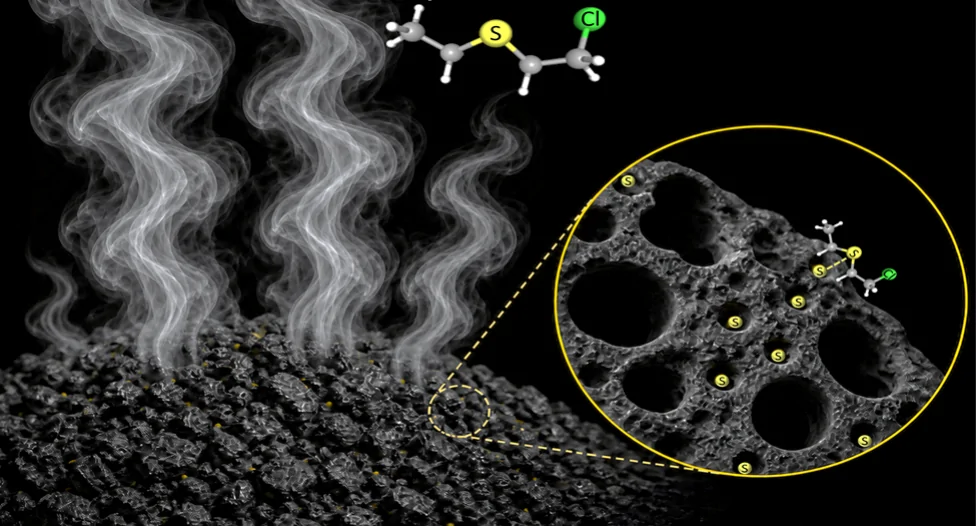

## 1 Introduction

Chemical warfare agents (CWAs) remain a significant global threat due to their extreme toxicity, environmental persistence, and potential deployment in armed conflicts, posing serious risks to both military personnel and civilian populations. Among these agents, bis­(2-chloroethyl) sulfide, also known as mustard gas, is recognized for its severe vesicant and cytotoxic properties, leading to an extensive blistering of skin, respiratory tract injury, and long-term carcinogenic and mutagenic effects.^1,2^ A major challenge in mitigating mustard gas exposure lies in its high chemical stability and pronounced lipophilicity, which allow the agent to persist on surfaces and penetrate most clothing.^3,4^ Its lipophilic nature also facilitates rapid infiltration through the skin and cellular membranes, increasing the risk of systemic toxicity.^3^ Consequently, effective remediation strategies not only must rely on adsorption of mustard gas but also should have the ability to chemically neutralize it into nontoxic products. This highlights the need for robust, field-deployable materials capable of both capturing and degrading CWAs under a variety of environmental conditions.^5–8^

The integration of carbon-based materials into personal protective equipment and filtration systems has been extensively reported as an effective strategy against CWAs.^7–11^ Activated carbons are widely used because of their availability, cost-effectiveness, and high adsorption capacity, especially for hydrophobic compounds. In addition, their large surface area and hierarchical porous structure, often extending to the nanoscale, contribute to the efficient capture of toxic compounds, maintaining a lightweight profile suitable for wearable applications.^7,8^ While the activity of pristine activated carbons primarily relies on physical adsorption, their surface chemistry can be tailored to promote both adsorption and catalytic degradation, allowing not only the capture but also the neutralization of hazardous compounds.^7^ Recent research has focused on optimizing pore architecture and introducing reactive surface functionalities to enhance the detoxification performance.^12–18^ Chemically modified activated carbons impregnated with basic inorganic compounds effectively degrade mustard gas into less harmful products through hydrolysis and elimination reactions.^12^ The effect of an oxidative treatment on carbon materials revealed that oxidized surfaces eliminate the mustard gas surrogate mainly through dehydrohalogenation while nonoxidized surfaces decompose the mustard gas surrogate via hydrolysis.^19^ Nevertheless, in all of these possible mechanisms for the removal of CWAs, the physical adsorption process still plays an important role, especially in high-porosity materials because they enable strong and rapid uptake of toxic compounds.^8^

In our recent study,^20^ sulfur was successfully incorporated into three distinct carbon hosts by using a steam-assisted sulfur insertion method. The resulting materials exhibited gradual differences in their surface features. Since the variation in porosity was one of the main goals of the surface tuning by sulfur, this study explores how the changes in the pore size and volume resulting from different sulfur loadings affect the adsorption of 2-chloroethyl ethyl sulfide (CEES) on the surface of porous carbons. The carbon surface was initially modified by introducing surface groups of different natures containing oxygen and/or nitrogen. Then, the predetermined amounts of sulfur were inserted into the porous structure to fill the pores gradually and coat the surface with a hydrophobic layer. The integrity of that layer depended on a defect population in graphene walls and was affected by carbon surface chemistry. Taking the above into account, this study aims to contribute to understanding the role of inserted sulfur not only in altering pore structure and surface chemistry but also in interactions with CEES in the confined space of carbon materials. It also analyzed how gradual changes in the pore size distributions and surface properties of sulfur-tuned carbons affected the extent and strength of CEES adsorption. Density functional theory (DFT) calculations were also performed to better understand the effect of sulfur on adsorption of CEES and its decomposition products. Since CEES is a small molecule of mainly hydrophobic properties,^21^ with a carbon–chlorine bond and thioether group contributing to its polarity,^22^ these findings may also provide insight into the adsorption and/or interactions of other organic molecules on modified carbon surfaces.

## 2 Materials and Methods

### 2.1 Preparation of Sulfur-Tuned Advanced Carbons

The detailed synthesis and characterization of sulfur-tuned modified carbons addressed in this study were reported elsewhere.^20^ In brief, a commercial carbon black (BP2000, Cabot) was modified through three distinct treatments: oxidation with hydrogen peroxide (STOC), oxidation with nitric acid (STONC), and a thermal treatment with urea (STUC). Each carbon host (0.25 g) was subsequently subjected to a hydrothermal treatment^23^ at 150 °C for 3 h in 50 mL of deionized water, using predetermined sulfur loadings corresponding to 0%, 5%, 10%, and 50% of the total pore volume of each carbon host. After being slowly cooled to a room temperature, the samples were separated by filtration and dried at 70 °C overnight.

Thermogravimetric profiles were recorded on a TA Instrument device (SDT Q600), at temperatures ranging from 50 to 1000 °C at a heating rate of 10 °C/min under a controlled flow of argon (100 mL/min). Simultaneously, the mass spectra of the released gases were analyzed using a mass spectrometer (ThermoStar, Pfeiffer Vacuum). The surface area and pore volume were evaluated from nitrogen adsorption isotherms measured on a Micromeritics ASAP 2020 instrument. Samples were outgassed overnight at 70 °C under vacuum and then analyzed at −196 °C. The pore size distributions were obtained using 2D-NLDFT (two-dimensional nonlocal density functional theory).^24^ Potentiometric titration was performed using 50 mg of carbon dispersed in 25 mL of 0.01 M NaNO_3_. After the sample was stirred overnight, the initial surface pH was recorded. The dispersions were titrated from pH 3 to 11 with NaOH (0.1004 M). The resulting proton binding curves were processed with the SAIEUS method to determine the p*K*_a_ distribution of the surface functional groups.^25^ X-ray photoelectron spectroscopy measurements were carried out on a VersaProbe II spectrometer (Physical Electronics PHI) using an Al Kα X-ray (50 W, 15 kV, 1486.6 eV) at a 45° angle on a 200 μm diameter analysis area.

### 2.2 CEES Adsorption Setup

All 2-chloroethyl ethyl sulfide adsorption experiments were performed in a closed system. Prior to the tests, all samples were dried at 70 °C overnight. For each experiment, 10 mg of carbon was placed in a 20 mL amber glass vial and 3 μL of liquid CEES (purity of >97%) was carefully dropped directly onto the carbon using a microsyringe. The vials were immediately sealed with septum caps and kept in the dark. After 24 h, 40 μL of vapor from the headspace was collected through the septum and analyzed by gas chromatography–mass spectrometry (GCMS). To evaluate the quantity of CEES and its reaction products retained on the carbon surface, an extraction was performed using 3 mL of high-purity acetonitrile (>99%, OmniSolv). The extracts were filtered through 0.22 μm nylon membranes, and 2 μL of the filtered solution was automatically injected into the GCMS instrument for quantification.

GCMS analysis was performed with an Agilent 7820A/5977B instrument, equipped with an Agilent HP-5MS ultrainert column (30 m × 0.25 mm × 0.25 μm). The oven temperature was set to increase from 50 to 110 °C at a rate of 5 °C/min and then increased to 270 °C at a rate of 40 °C/min. Helium was used as the carrier gas at a flow rate of 33.7 mL/min. Split ratios were adjusted to 10:1 and 50:1 for samples from headspace and extracts, respectively.

### 2.3 Energy of Interactions

Differential scanning calorimetry (DSC) measurements were performed on a microcalorimeter (Microcalvet, Setaram). Twenty-five milligrams of adsorbent were introduced into the lower chamber of a closed mixing vessel, and the upper chamber was filled with a predetermined volume of CEES corresponding to the total pore volume of each carbon sample. After stabilization at 20 °C, the upper chamber was opened to allow contact between CEES and the adsorbent, and the change in heat flow to keep the vessel at 20 °C was measured. The energy of interactions was calculated by integrating the heat flow curves. To account for the effects of the amounts of CEES used in the tests, the results were normalized to these values.

### 2.4 Computational Details

Adsorption of CEES, DEDS, 2CE, and HEES and the associated energetic effects were studied with the aid of density functional theory (DFT) with the B3LYP exchange-correlation functional.^26^ The D3 empirical correction^27^ was included to account for dispersive interactions. Calculations were performed with Pople’s 6-311G** basis set.^28^ This computational level is adequate to provide insight into processes that may occur in pores, providing a reasonable compromise between the quality of the results and computational time.^29^

Interaction energies (IEs) related to physical adsorption were calculated within the supermolecular approach as negative values of *E*_product_ – *E*_substrates_, where *E*_substrates_ refers to the sum of the total energies of the relevant adsorbate and a representative fragment of graphene (alone or functionalized with OH or COOH groups, with or without the adsorbed sulfur species (*vide infra*)), and *E*_product_ refers to the total energy of the complex of CEES, DEDS, 2CE, or HEES with the (sulfurized) graphene fragment. Thus, the higher the IE value, the greater the stabilization of the system. Note that IE values are based on energies of molecules having geometries as in a complex, i.e., distorted from equilibrium (due to interactions between adsorbent and adsorbate molecules). This implies that for an AB system an IE = *E*_AB/AB_ – *E*_A/AB_ – *E*_B/AB_ formula was used (the subscript .../AB indicates that the geometries of A, B, and AB found for the equilibrium geometry of the AB complex were adopted). The methodology behind the choice of the appropriate representative fragment was described elsewhere.^30,31^ The IE values were corrected for a basis set superposition error (BSSE) using a counterpoise correction scheme (CP).^32^ They are denoted IE+BSSE; note that they are by far more reliable than raw IEs. The equilibrium geometries and harmonic vibrational frequencies were obtained for each structure first. If all frequencies were real, a system was considered to be stable. All systems, surfaces, and adsorbed molecules were closed-shell singlets. All calculations were performed using the Gaussian 16 quantum chemistry package.^33^

## 3 Results and Discussion

### 3.1 Surface Characterization

The main surface features of the samples are summarized in Figure 1. The contribution of ultramicropores (<0.7 nm), supermicropores (0.7–2 nm), and mesopores (>2 nm) to the total pore volumes (Figure 1a) and the surface area in pores of different sizes (Figure 1b) show the heterogeneity of the pore structure in all three carbon hosts. Among the samples, STUC-0 exhibited the largest total pore volume (2.69 cm^3^/g) and surface area (1476 m^2^/g). The STOC-0 porosity parameters (*V*_T_ = 2.45 cm^3^/g; *S*_BET_ = 1372 m^2^/g) were similar to those of STUC-0, while STONC-0 showed the smallest total pore volume (1.65 cm^3^/g) and surface area (1194 m^2^/g).^20^ STONC-0 also had the largest volume of ultramicropores and the smallest volume of mesopores. The introduction of sulfur resulted in a gradual decrease in the surface area and total pore volume for all series of carbons. At loadings of 5% of *V*_T_, sulfur insertion caused the smallest decrease in the surface area and pore volume, with sulfur occupying mainly the ultramicropores. When the loading reached 10% of *V*_T_, all ultramicropores in STOC-10 and STUC-10 were filled. STONC-10 still retained some ultramicroporosity due to the initially larger fraction of these pores. Increasing the sulfur content to 50% of *V*_T_ resulted in the complete filling of the micropores in all samples, leaving only a small volume of larger mesopores unoccupied.

**Figure 1:**
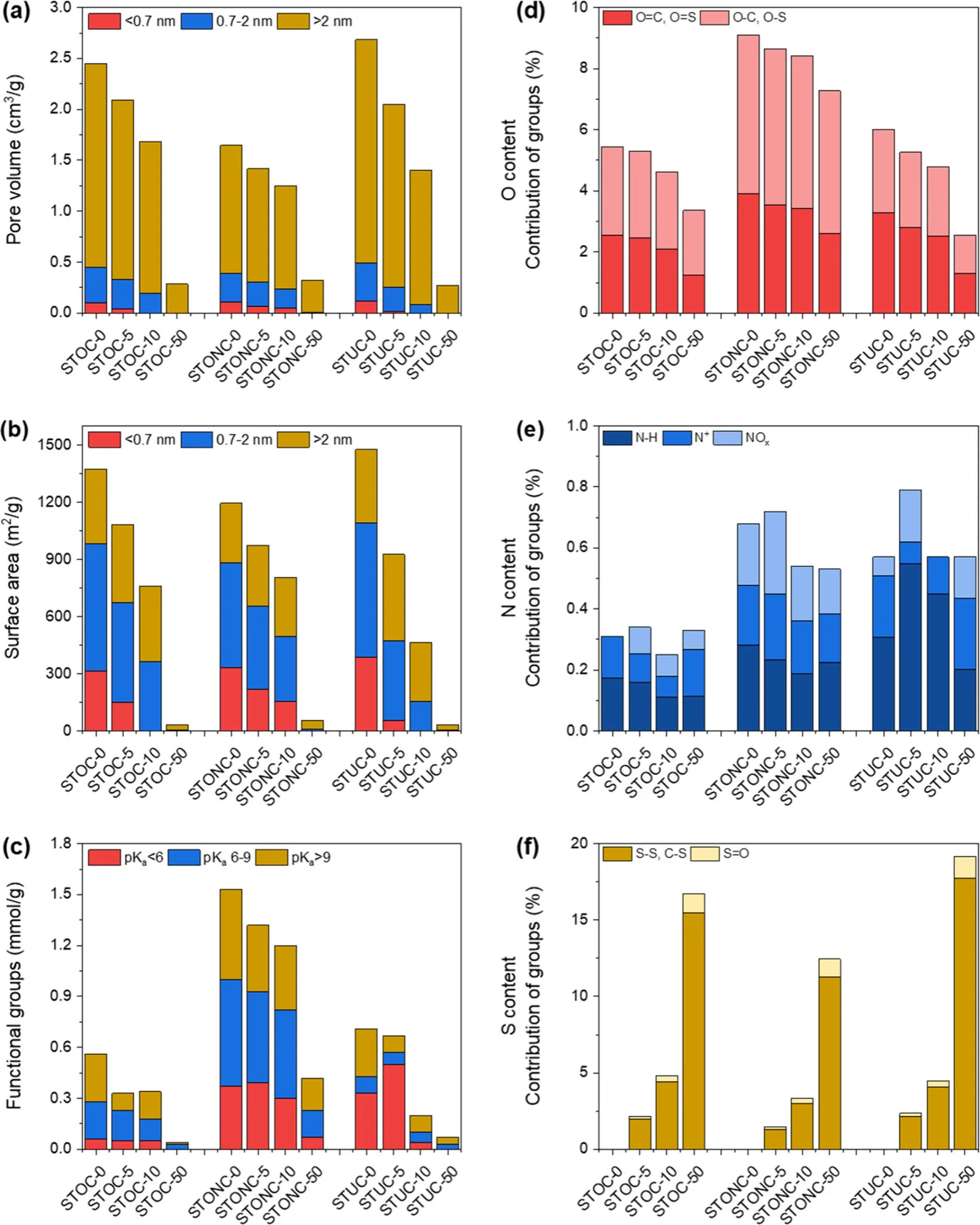
(a) Contributions of specific pore volumes. (b) Contributions of the surface area in pores of specific sizes. (c) Surface functional groups categorized based on their p*K*_a_ values. (d) Contributions of specific carbon–oxygen bonds to deconvoluted O 1s spectra. (e) Contributions of specific nitrogen-containing surface groups to deconvoluted N 1s spectra. (f) Contributions of specific sulfur-containing surface groups to deconvoluted S 2p spectra.

The initial surface pH values of the carbon surfaces were 3.2, 5.2, and 7.2 for STONC-0, STOC-0, and STUC-0, respectively.^20^ These differences reflect the extent of the oxidation treatment, which increased the acidity of STONC-0 and STOC-0 compared to that of initial carbon black whose pH was 8.0.^34^ The high-temperature treatment of the initial carbon black in the presence of adsorbed urea (STUC-0) increased its basicity through the introduction of nitrogen and surface reduction upon heating in an inert atmosphere.

Figure 1c provides the contents of surface functional groups with various p*K*_a_ values (strongly acidic, close to neutral, and weakly acidic). STONC-0 had the largest number of functional groups (1.53 mmol/g), followed by STUC-0 (0.71 mmol/g) and STOC-0 (0.56 mmol/g). Those groups on STONC-0 were also the most acidic. Notably, the acid–base properties of the two oxidized hosts were similar to sulfur loadings of 5% and 10% of *V*_T_ and showed a decrease in acidity for the samples with a sulfur loading of 50% of *V*_T_. This trend suggested that oxygen-containing groups were predominantly located in the larger pores. The relative content of the dissociating groups of different strengths of the STONC series was most affected with an increase in the number of strongly acidic groups, likely carboxylic, for the sample with 5% of pore filling by sulfur. This was associated with a marked decrease in the number of weakly acidic groups, likely phenolic, suggesting the location of basic groups also in small pores and a higher extent of oxidation of deposited sulfur than in other samples. That latter scenario might be linked to the well-known activation of oxygen by nitrogen groups on the carbon surface resulting in the formation of ROS.^35^ When 10% of the pore volume was filled by sulfur in STUC, the contribution of strongly acidic groups markedly decreased along with a significant decrease in the amount of all dissociating groups, suggesting differences in the surface distribution of groups between this sample and its other two counterparts. Another plausible explanation might be in the higher level of integrity of the sulfur lining that covers the hydrophobic surface of STUC, as opposed to the existence of detached sulfur patches on more hydrophilic oxidized surfaces of the STONC samples. Support for this is found in the trend in the density of dissociating groups, which was calculated assuming their location on the surface in mesopores (Table S1). The most marked decrease in the density of these groups between samples with 0% and 10% of *V*_T_ was for the STUC series (from 1.8 × 10^–3^ to 0.7 × 10^–3^ mmol/m^2^). For STOC, the change was from 1.4 × 10^–3^ to 0.9 × 10^–3^ mmol/m^2^, and for STONC, it was from 4.9 × 10^–3^ to 3.9 × 10^–3^ mmol/m^2^. At 50% pore filling with sulfur, still a high density of groups was found for STONC-50 (9.6 × 10^–3^ mmol/m^2^) followed by STUC-50 (2.3 × 10^–3^ mmol/m^2^) and STOC-50 (1.3 × 10^–3^ mmol/m^2^), supporting the high level of integrity of the sulfur lining on the latter two samples.

XPS analysis showed that the oxygen content was 5.5 at.% for STOC-0, 9.1 at.% for STONC-0, and 6.0 at.% for STUC-0, with the presence of C=O and C–O functional groups on the surface of all samples (Figure 1d). The nitrogen content was 0.3 at.% for STOC-0, 0.7 at.% for STONC-0, and 0.6 at.% for STUC-0 (Figure 1e). That nitrogen was mainly in N–H, N-quaternary, and NO_*x*_ functional groups. The latter was predominant on the HNO_3_-oxidized sample, increasing the surface polarity. After sulfur was inserted, the nitrogen content remained similar across the samples; however, a decrease in oxygen content was observed with an increase in sulfur loading. For the STOC series, it gradually decreased from 5.3 at.% in STOC-5 to 4.6 at.% in STOC-10 and reached 3.4 at.% in STOC-50. For STONC, the changes were from 8.6 at.% in STONC-5 to 8.4 at.% in STONC-10 to 7.3 at.% in STONC-50. The most pronounced changes, like those in the density of the dissociating groups, were for the STUC series (5.3 at.% in STUC-5, 4.8 at.% in STUC-10, and 2.6 at.% in STUC-50).^20^ Interestingly, the introduction of 5% and 10% sulfur only slightly affected the oxygen content and the distribution of groups, implying that the applied chemical treatment affected the surface in large pores and/or mesopores, which were not filled at those loadings. With the insertion of sulfur, its amount gradually increased across all three series (Figure 1f), reaching 2.2 at.% in STOC-5, 4.8 at.% in STOC-10, 16.7 at.% in STOC-50, 1.5 at.% in STONC-5, 3.4 at.% in STONC-10, 12.5 at.% in STONC-50, 2.4 atom % in STUC-5, 4.5 at.% in STUC-10, and 19.2 at.% in STUC-50.^20^ Most of the detected sulfur exists in an elemental state (S^0^), although contributions from C–S and C–S–S–C bonds cannot be excluded because their binding energies overlap with that of elemental sulfur.^36,37^ A minor presence of oxidized sulfur species (S=O) was also found in all sulfur-modified samples, and their concentrations increased

### 3.2 Surface Interaction between the Carbon Surface and CEES

To assess CEES adsorption on the studied carbon samples, we thoroughly analyzed both headspace vapors and acetonitrile extracts from the exposed materials. The identified compounds, along with their abbreviations and chemical formulas, are listed in Table 1.

**Table 1:** Compounds Detected in the Headspace and Extracts

compound	abbreviation	chemical formula	MW
2-chloroethyl ethyl sulfide	CEES	CH_3_CH_2_SCH_2_CH_2_Cl	124
dichloroethane	2CE	ClCH_2_CH_2_Cl	98
diethyl disulfide	DEDS	CH_3_CH_2_SSCH_2_CH_3_	122
hydroxyethyl ethyl sulfide	HEES	CH_3_CH_2_SCH_2_CH_2_OH	106
1,2-bis(ethylthio)ethane	BETE	CH_3_CH_2_SCH_2_CH_2_SCH_2_CH_3_	150
1-chloro-2-([2-(ethylsufanyl)ethyl]sulfanyl)ethane	CESESE	CH_3_CH_2_SCH_2_CH_2_SCH_2_CH_2_Cl	184
bis[2-(ethylthio)ethyl] ether	BETEE	CH_3_CH_2_SCH_2_CH_2_OCH_2_CH_2_SCH_2_CH_3_	194

Figure 2 presents the results of the GCMS analysis of the headspaces of the test vials and the extracts from carbons after exposure for 24 h to CEES. As expected, the amounts of CEES vapors existing in the headspace in the presence of carbons were much smaller than in the blank test (Figure 2a). The ability of the modified carbons to physically adsorb the liquid and inhibit CEES vaporization decreased with an increase in sulfur loading across all carbon series, which can be linked to the decrease in porosity and available surface area. For instance, a significantly larger quantity of CEES vapors detected in the headspace of the STUC-10 vial compared to those for the STOC-10 and STONC-10 counterparts is likely due to its much smaller surface area (463 m^2^/g), which was 39 and 43% smaller than those of STOC-10 (761 m^2^/g) and STONC-10 (806 m^2^/g), respectively. However, STOC-10 released much less CEES vapor than did STONC-10, despite very small differences in the surface area between these two samples. The main difference between these two carbons is in the sulfur content in terms of weight (less on STOC-10 due to its smaller porosity) and in the population of dissociating groups on the surface. The latter might affect the uniformity of the sulfur lining. More than 3 times more groups on STONC-10 than on STOC-10 might lead to a more patchwise nature of that lining on the surface of the former sample than on the latter, and this might result in the weaker interactions of CEES with the STONC-10 surface.

**Figure 2:**
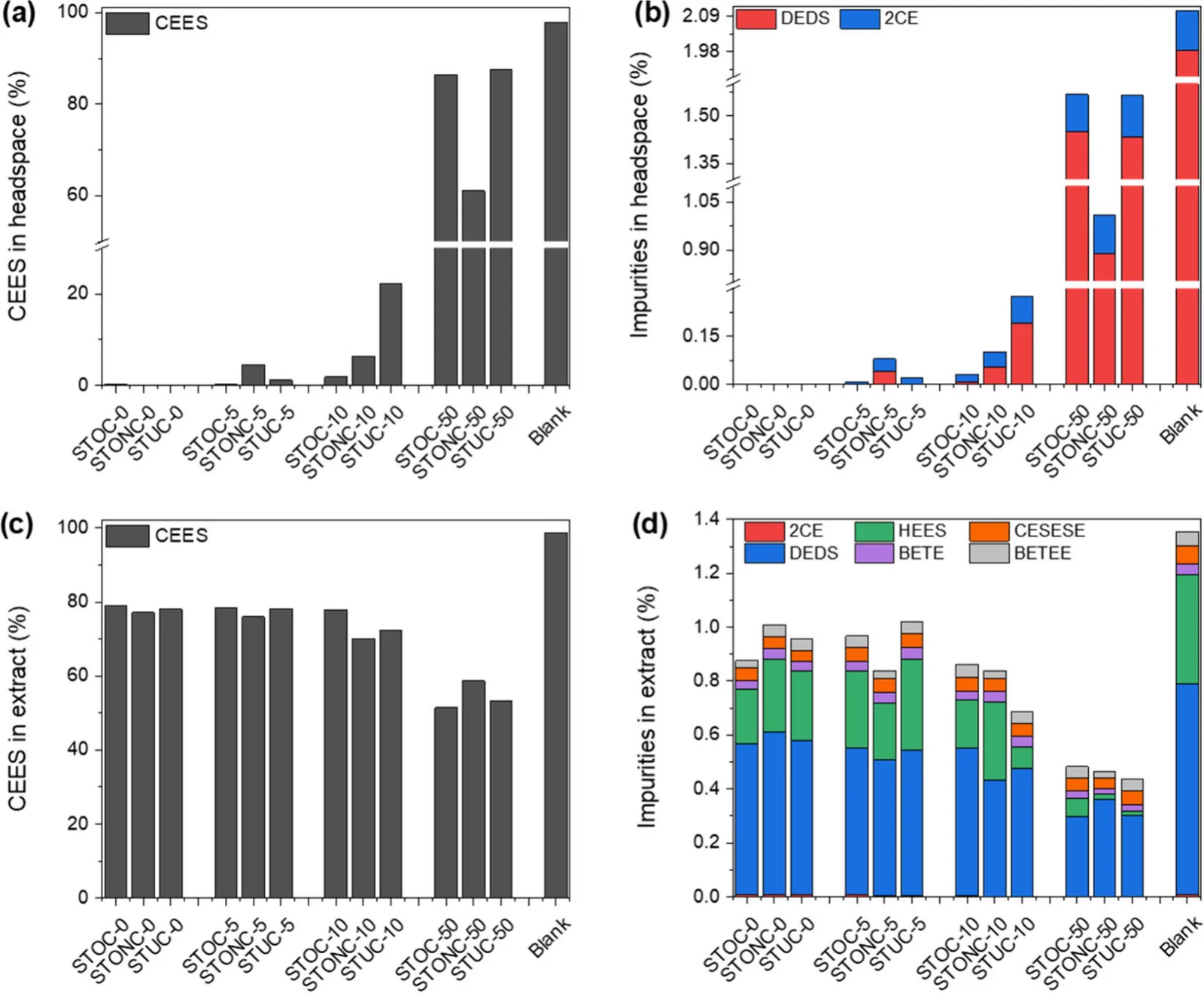
Amounts of CEES and other detected compounds in the (a and b, respectively) headspace and (c and d, respectively) extracts from droplet tests.

In addition to CEES, traces of impurities, 2CE and DEDS, were detected in the headspace of the blank test (Figure 2b). The amounts of these impurities decreased after contact with the tested carbons, which is likely due to their adsorption in the pores of the materials. On the samples without sulfur, whose porosities were the highest, both 2CE and DEDS were completely adsorbed. Traces of 2CE remained in the headspace of STOC-5, STONC-5, and STUC-5, but DEDS was fully adsorbed by STOC-5 and STUC-5, which might be attributed to the stronger adsorption of the large DEDS molecule than that of the smaller 2CE molecule. Also, S–S bonds in the DEDS molecule could provide more affinity to elemental sulfur deposited on the carbon surface. The calculated energies of the interactions are discussed below. Even though the porosity of STOC-10 was slightly lower than that of STONC-10, it removed more CEES and DEDS than the latter sample, and this trend could be also due to the differences in the integrity of the sulfur lining discussed above. Moreover, STONC-10 still has a significant number of surface acidic groups, which provide hydrophilicity to the carbon surface and steric hindrance, decreasing the adsorption of both DEDS and CEES. Interestingly, reaction products were not detected in the headspace of any modified carbon, implying a lack of surface reactivity of carbon blacks, which is unusual compared with other porous carbons.^7,8,12,17,18^

Figure 2c presents a comparison of the amounts of CEES detected in the acetonitrile extracts from the spent carbon samples. In this case, the blank test corresponds to the composition of the CEES stock solution, indicating traces of other impurities (less than 2%). After contact with carbon, the amounts of CEES extracted from all tested samples were smaller than the amount detected in the blank test. This decrease could be a combined result of both the strong retention of CEES on the surface and CEES volatilization and thus its presence in the headspace. Figure S1 further supports this by showing an inverse correlation between the quantity of CEES vapors detected in the headspace and the amounts of CEES extracted from the exhausted carbons (*R* ^2^ = 0.96).

Figure 2d shows that the amounts of detected impurities, 2CE, DEDS, HEES, BETE, CESESE, and BETEE, also decreased after contact with the modified carbon samples compared to those in the blank test. However, the fraction of each detected compound varied among the samples. As shown in Figure 2d, the amounts of impurities extracted from exposed STOC-50, STONC-50, and STUC-50 were significantly smaller, which can be attributed to the large amounts of vapors released to their headspaces (Figure 2b). The fraction of HEES significantly varied among the samples, and it decreased significantly in the extracts from STOC-50, STONC-50, and STUC-50, compared to the samples with less sulfur. This implies that the presence of sulfur promotes HEES adsorption. Interestingly, a small quantity of HEES was detected for STUC-10 compared to those for STOC-10 and STONC-10, which has the smallest surface area. The species that could promote the adsorption of HEES on oxidized carbons via specific interactions could also be the polar groups attracting the OH moiety of HEES, and the energy of these interactions is discussed below. Moreover, nitrogen groups providing hydrogen bonds might also play a role.

To roughly evaluate the extent of CEES desorption from the samples tested, thermal analysis was performed on the spent samples and those after extractions. The results are compared to those on the initial ones (Figure 3). Semiquantitative information about the strength of CEES adsorption can be unveiled by analyzing the differences in the amounts of CEES adsorbed and retained after extraction. For the sake of clarity, the results are presented in columns corresponding to samples without sulfur (a–c), and with sulfur loadings of 5% of *V*_T_ (d–f), 10% of *V*_T_ (g–i), and 50% of *V*_T_ (j–l). Rows a–c represent the STOC, STONC, and STUC series, respectively. The peaks on the DTG curves correspond to the weight loss events in specific temperature ranges standing for the decomposition of surface groups and/or removal of the adsorbed species due to applied heat. The functional groups of STOC-0 and STUC-0 are removed in the limited temperature range between 200 and 300 °C. After adsorption of CEES, well-defined peaks appeared between 150 and 400 °C, and they represent the removal of CEES. This process is more complex for STOC-0 and STUC-0, in which three shoulders can be distinguished around 180, 220, and 300 °C. They are linked to the removal of CEES from distinct and increasing-in-strength adsorption centers that are likely pores of different sizes. The removal of CEES from smaller pores requires more energy, and the peaks are shifted to higher temperatures.^38^ Mass spectroscopy analysis (Figures S2–S4) revealed that between 100 and 400 °C, ions at *m*/*z* 27 and 35 are detected representing C_2_H_3_ fragments of the CEES molecule and chlorine, respectively.

**Figure 3:**
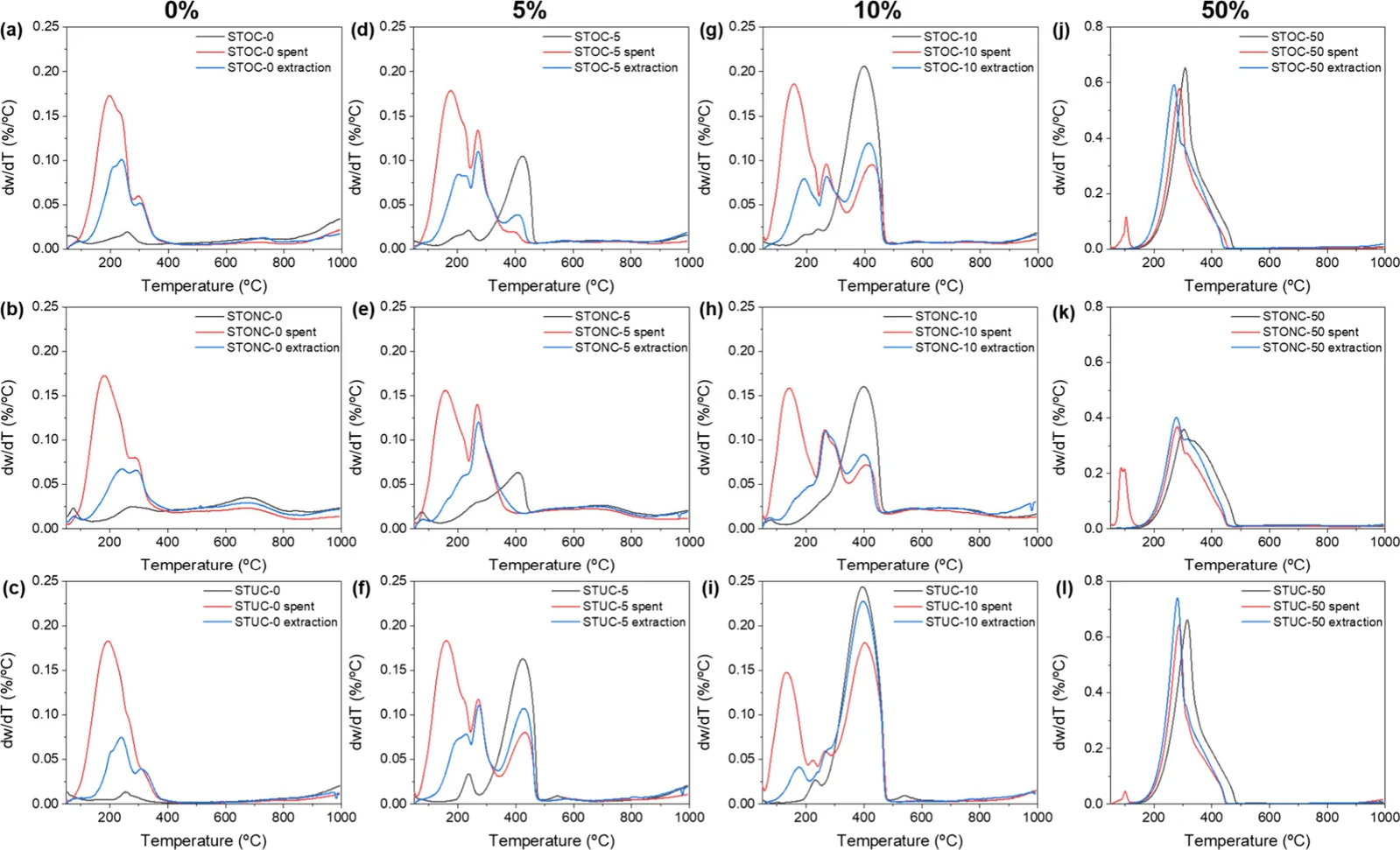
Comparison of DTG curves measured in argon for the samples with (a–c) 0%, (d–f) 5%, (g–i) 10%, and (j–l) 50% of the total pore volume filled with sulfur.

The differences in the weight loss between the spent samples and those after extraction showed that the percentages of CEES extracted were 42%, 51%, and 59% for STOC-0, STONC-0, and STUC-0, respectively, as compared to those adsorbed on the spent samples. No dependence between the amount extracted and the volume of pores in all categories was found, suggesting that carbon surface chemistry might play a role in CEES retention. Interestingly, the highest efficiency of extraction was found for STUC-0, which is the most porous sample and the most basic one. The weight loss patterns for the samples after extraction revealed an increase in diversity and in the strength of the adsorption centers. While the weakly adsorbed CEES was removed to a great extent from all samples, some part of it was still retained on up to three distinct energy centers.

The deposition of sulfur distinctively changed the thermal decomposition patterns of the tested carbons. The removal of sulfur during heating from the unexposed samples corresponding to sulfur loadings of 5% of *V*_T_ occurs between 200 and 400 °C. The highest temperature of removal can be linked to the highest extent of penetration of sulfur into the smallest pores, where its adsorption is the strongest.^38,39^ However, in those pores, sulfur fragments are rather smaller than the S_8_ rings are, due to the steric constraint of the pore sizes.^32^ The weight loss at about 200 °C is linked to both the removal of sulfur from the external surface and the decomposition of the surface groups, since only a small fraction of pores was filled and functional groups are still present on the surface (Figure 3). Upon adsorption of CEES on those samples, the weight loss patterns associated with the removal of adsorbed species increased in complexity and a sharp peak of nonextractable species appeared at ∼260 °C. Based on the lack of the weight loss event representing the removal of sulfur at about 400 °C from the smallest pores of STONC-5 exposed to CEES, one can speculate that these pores are not accessible to sulfur due to the steric obstacles of surface functional groups. In the case of all samples with 5% filling of their pores with sulfur, the *m*/*z* fragments representing H_2_S, COS, and CS_2_ (Figure 4) were detected between 250 and 300 °C likely as a result of the reactions of CEES with the carbon surface during heating. Since the carbon surface, especially that of STONC-5, is rich in oxygen and sulfur exists there as small reactive fragments, it is possible that at this temperature the complex bond breaking and redox reactions take place. The efficiencies of extraction calculated from the weight loss below 350 °C were 41%, 43%, and 52% for STOC-5, STONC-5, and STUC-5, respectively. Since the inverse of the extraction efficiency reflects the quantity of CEES retained in the pores, plotting the dependence of this factor on the volume of micropores showed the linear trend with *R* ^2^ = 0.98 (Figure 5a), emphasizing the role of micropores in CEES adsorption.

**Figure 4:**
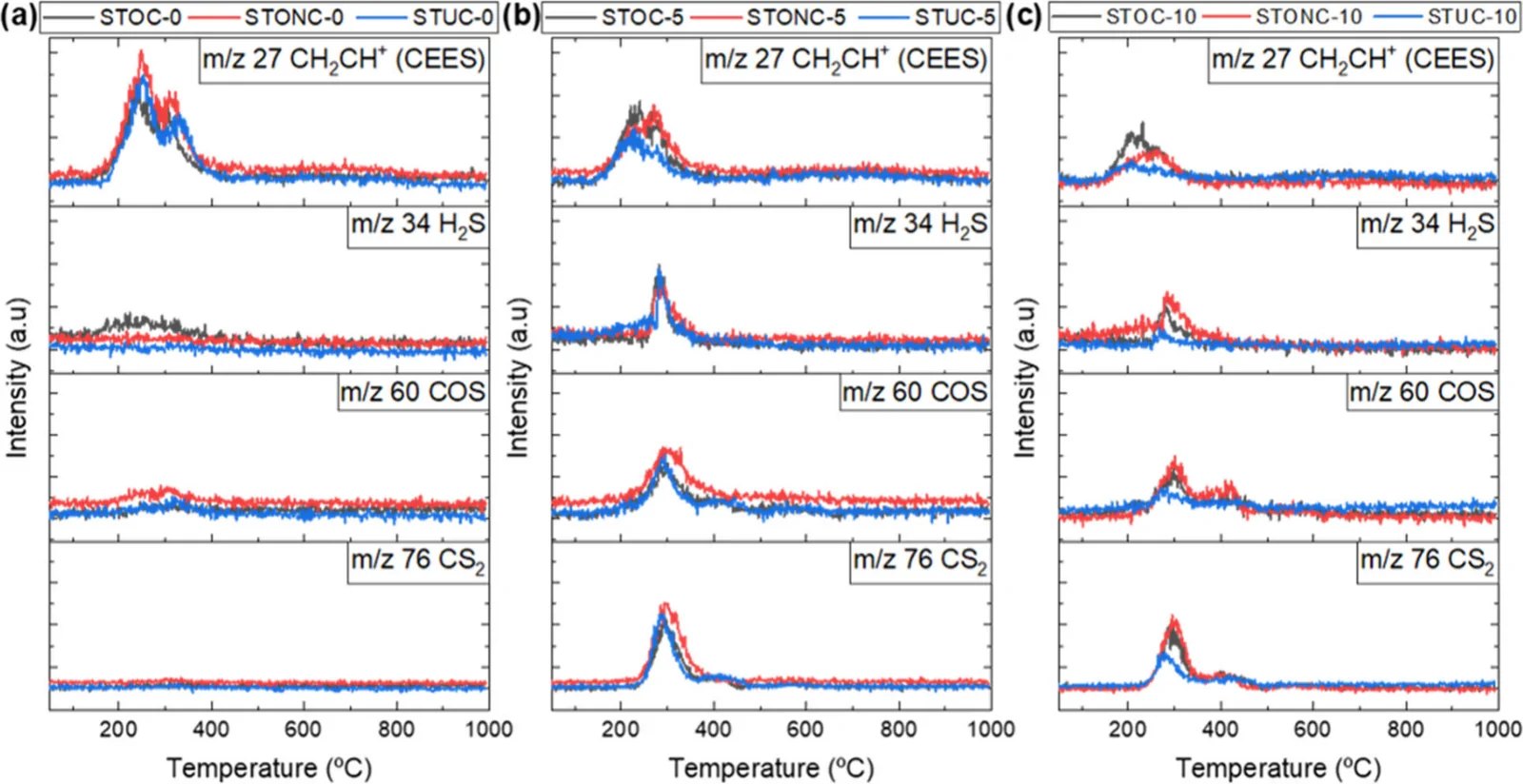
Thermal profiles of *m*/*z* fragments corresponding to DTG curves for the samples after extraction: (a) the sulfur-free samples and samples (b) with 5% of *V*_T_ filled with sulfur and (c) with 10% of *V*_T_ filled with sulfur.

**Figure 5:**
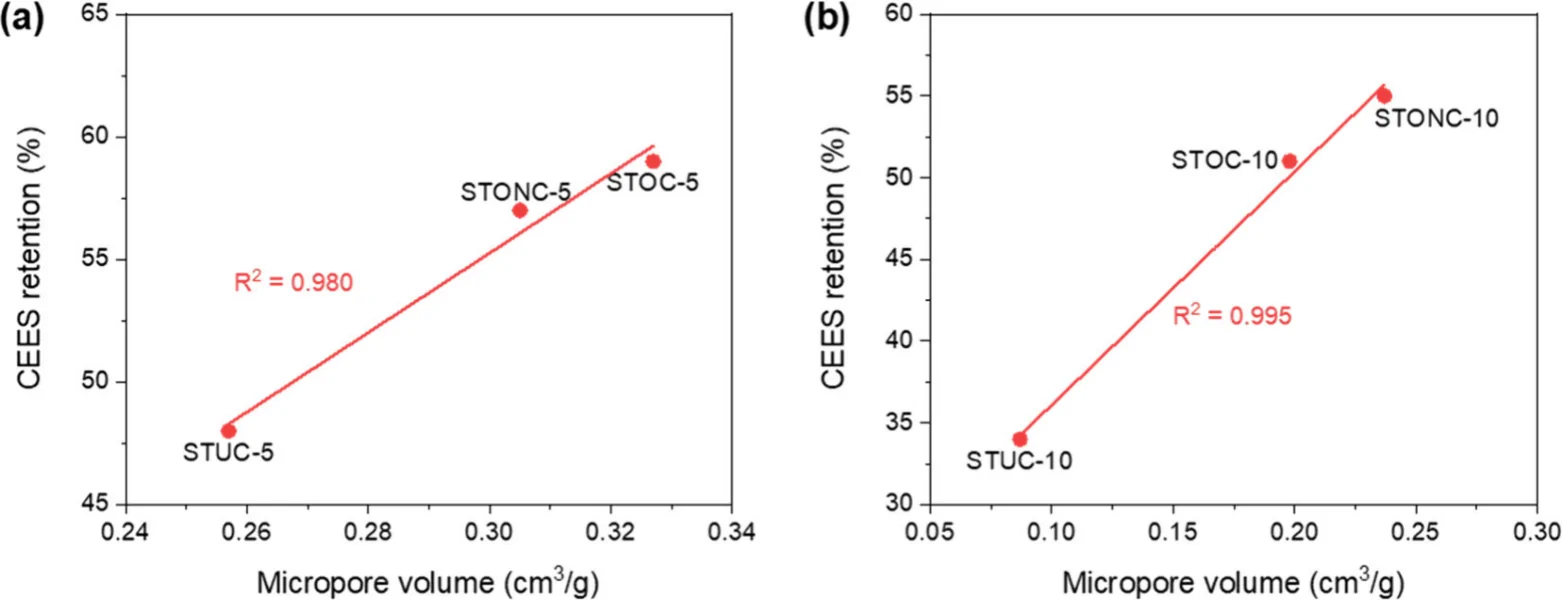
Dependence between the percentage of CEES retained on the sample surface after extraction and the volume of micropores at sulfur loadings corresponding to (a) 5% of *V*_T_ and (b) 10% of *V*_T_.

The samples with 10% of the total pore volume filled with sulfur show more changes in the thermal stability patterns of CEES-exposed carbons than those with 5% sulfur. Their main weight loss events appear at 180 and 220 °C. However, the latter almost does not exist for STUC-10. The species that decompose at 220 °C remain unchanged after extraction. The efficiencies of extraction in this case were 49%, 45%, and 66%, for STOC-10, STONC-10, and STUC-10, respectively, showing STONC-10 as the strongest adsorbing sample. The reason for this might be that 42% of the original volume of ultramicropores remains in STONC-10. This implies that sulfur there is present in the smallest range of pore sizes that have rather a hydrophobic surface and where CEES retention is the strongest. In the case of STUC-10, these pores are completely filled and/or clogged by sulfur. For the 10% series, the dependence of the CEES retained on the volume of the micropores (Figure 5b) also shows a linear trend with *R* ^2^ = 0.99, supporting the adsorption process in these pores where sulfur and carbon surfaces coexist.

With 50% pore filling, the surface area is rather small leading to a very small quantity of CEES adsorbed. The order of weight loss found for these samples (STONC-50 < STOC-50 ≈ STUC-50) directly correlates with the quantities of CEES detected in the headspace, as demonstrated by *R* ^2^ = 0.975 in Figure S5. Interestingly, for these samples, a peak at 100 °C appears and its intensity follows both the oxygen content on the surface and the available porosity. The mass spectroscopy results indicated the release of Cl, CSO, C_2_H_3_, and CS_2_ (Figures S2–S4). These species did not appear for the extracted samples. They might represent CEES adsorbed on the external surface, and the temperature of removal corresponds to that vaporization of pure CEES (Figure S6). However, the detection of CSO implies some reactivity of CEES with the carbon functional groups upon heat treatment.

### 3.3 Energetics of Interactions

To further study the effects of carbon features on CEES adsorption, differential scanning calorimetry experiments were performed in which the heat released upon interactions of CEES with the carbon surface was measured. Measured heat flow curves are collected in Figure S7. It is worth mentioning that to obtain meaningful results and to address the instrument sensitivity, a volume of CEES, corresponding to 100% of *V*_T_ of each carbon, was injected into the testing cell to fill the pores. This is more than that in the case of droplet tests. The results were normalized taking into account the amount of CEES in the pore system (Figure 6). Pure CEES was also injected into an empty cell (no adsorbent), resulting in the expected endothermic peak corresponding to its vaporization (Figure S7). In contrast, when CEES interacted with STOC-0 or STUC-0, a sharp release of heat was initially observed with a relatively fast decrease during the period from 5 to 10 min, followed by a long tail. This indicated a good affinity of CEES for the STOC-0 and STUC-0 surfaces. The surface of STONC-0, on the contrary, appeared to be less attractive to CEES. Indeed, upon injections of CEES into the measurement cell, a small endothermic effect related to CEES evaporation was first recorded, and it could represent the difference between the exothermic effects of adsorption and that of vaporization (Figure S7), as a result of the two competing processes. This indicates that some vaporization of CEES is still happening, and it is faster than the adsorption process on STONC-0. After 6–8 min, an exothermic peak appeared, indicating that the bulk of adsorption occurs with a delay, which could be due to the low wettability of the STONC-0 surface by CEES. The samples containing 5% sulfur present the same behavior, but with a much less intense exothermic peak, indicating the much lower heat of interactions when the smallest pores are filled with sulfur.

**Figure 6:**
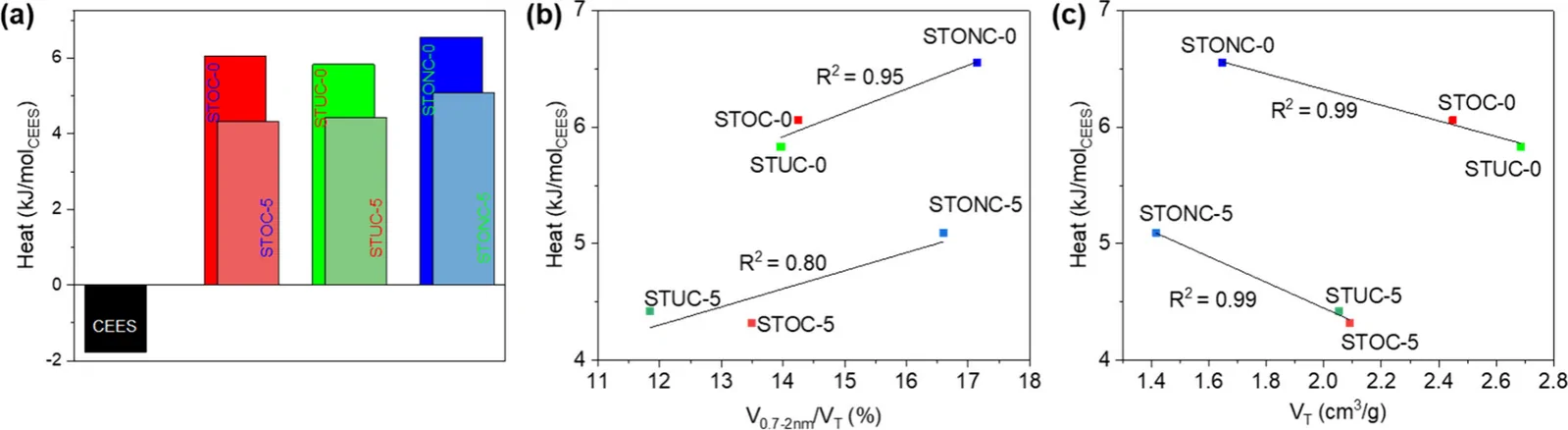
(a) Energies of interaction based on the integration of heat flow curves measured for sulfur-tuned carbons loaded with 0% and 5% of *V*_T_. (b) Dependence of the released heat on the degree of microporosity. (c) Dependence of the released heat on the total pore volume.

The calculated total energies of interactions (by integration of the heat flow curves) normalized per quantity of CEES added (corresponding to the amount needed to fill 100% of *V*_T_) are compared in Figure 6a. The initial STOC-0 and STUC-0 have the smallest amount of released heat per mole of CEES adsorbed in the pore system, with the latter exhibiting slightly weaker interactions. STONC-0 released about 10% more energy than other samples. The energies of interaction for STOC-0, STUC-0, and STONC-0 were 6.1, 5.8, and 6.6 kJ/mol, respectively, inversely following the trend in the surface area (Figure S8). The larger surface area is usually linked to larger pores, where adsorption is the weakest. A direct correlation with *R* ^2^ = 95 was found between the normalized heat of adsorption and the degree of microporosity (*V*_0.7–2 nm_/*V*_T_), stressing the importance of the latter in the extent of the energetics of adsorption.

After the introduction of sulfur corresponding to 5% of *V*_T_, the total energies of interactions decreased by 29%, 24%, and 22% for STOC-5, STUC-5, and STONC-5, respectively (4.3, 4.4, and 5.1 kJ/mol, respectively). The most important reason for this decrease is a decrease in the number of the smallest pores of the highest energy, which are now occupied by sulfur. Even though for these series of samples there is no good linear correlation between the heat of adsorption and the degree of microporosity (Figure 6b), the energy of interactions is still the highest for the samples with the highest *V*_0.7–2 nm_/*V*_T_ (STONC-5). Interestingly, the energies of interaction for STOC-5 and STUC-5 are very close to each other (4.42 ± 0.1 kJ/mol) with the degree of microporosity of the latter sample being 20% lower than that of the former. This might suggest that deposited sulfur with a high degree of integrity of its lining on the surface of STUC-5 contributes to the increase in the dispersive CEES–surface interactions. Even though for both series of samples (initial and those with 5% of total pore volume filled with sulfur) inverse relationships of the released heat with the total pore volume (larger pore volume = larger pores = weaker interactions) were found, the slope for the sulfur-filled samples was larger, suggesting an increase in the dispersive interactions of CEES with the surface due to the presence of sulfur in the pores of these samples.

### 3.4 DFT Results

A relatively large representative fragment of graphene (56765 fragment (Figure 7a)) was used in all calculations. Upon modification of porous carbons by sulfur insertion, the sulfur molecules interact with the graphene layer mostly via quadrupole–quadrupole (*U* ^(int)^ ∼ *r* ^–5^, where *r* denotes the distance between interacting species) and dispersive (*U* ^(int)^ ∼ *r* ^–6^) interactions. The latter ones are dominant at short distances due to the slightly higher polarizability of sulfur compared to that of carbon. The relatively large S_8_-ring molecules enter ultramicropores as S_*x*_ fragments^20,40,41^ (e.g., planar and outspread tetramers). Thus, a variety of stable surface–sulfur structures, an example of which is shown in Figure 7b, can be formed. Indeed, an overall energy increase associated with the breaking of S_8_ sulfur rings is compensated by the favorable interactions of these fragments with the graphene surface. The calculated BSSE-corrected interaction energy of (S_4_)_3_ with the graphene fragment is 49.16 kcal/mol (Table S2; hereafter, the BSSE-corrected interaction energy values will be reported since they are by far more reliable than uncorrected energies). That high value implies the stronger tendency of sulfur to form a continuous and high-structural integrity lining on the surface of carbons with fewer functional groups as opposed to that with a large population of groups, especially those of hydrophilic character. The stable configurations of CEES and DEDS are formed on pristine graphene with interaction energies of 15.81 and 14.19 kcal/mol, respectively (Figure 8), which are shown in panels c and d, respectively, of Figure 7. They are not expected to change much for different molecule–surface arrangements. The lowest interaction energy was obtained for 2CE (10.67 kcal/mol (Figure S9a and Figure 8)). The energy of interaction of HEES with the graphene surface (15.03 kcal/mol (Figure S9b and Figure 8)) is comparable to those found for CEES and DEDS, but additional interactions occur on functionalized graphene due to hydrogen bond formation (19.74 kcal/mol (Figure S9c)).

**Figure 7:**
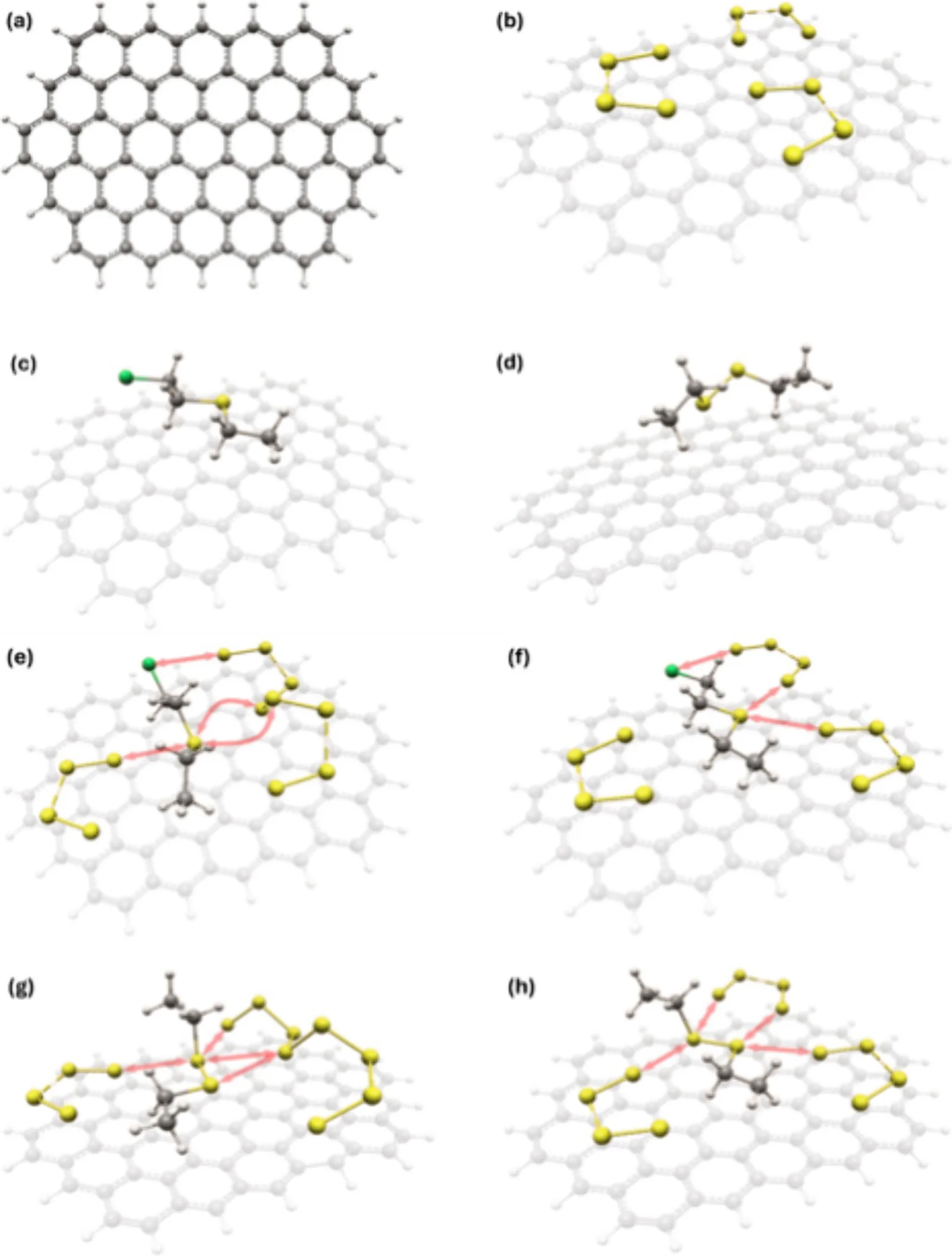
(a) Representative graphene fragment 56765 used in the calculations. (b) Example of a graphene–sulfur complex. Examples of complexes of (c) CEES and (d) DEDS with pristine graphene. Examples of complexes of (e and f) CEES and (g and h) DEDS with sulfurized surfaces. The red arrows indicate S_surface_–S_CEES_, S_surface_–Cl_CEES_, and S_surface_–S_DEDS_ interactions, in which internuclear distances are in the range of 3.2–3.8 Å.

**Figure 8:**
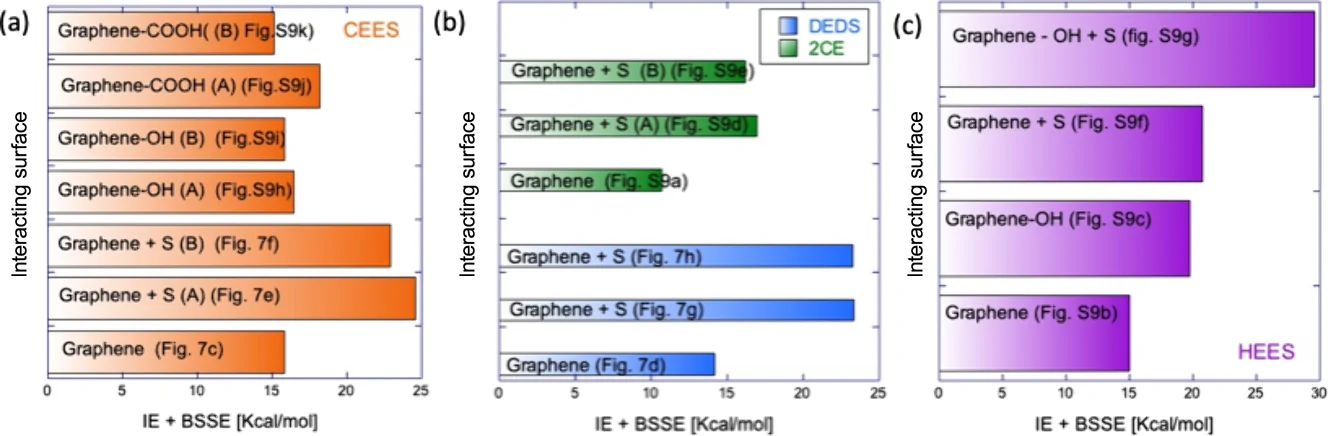
Comparison of the energies of interaction of (a) CEES, (b) DEDS and 2CE, and (c) HEES with the modified graphene surfaces. Specific configurations are presented in Figure 7 and Figures S8 and S9.

At a low sulfur loading, small sulfur structures (like tetramers) might “slide” along the graphene surface forming numerous local minima linked via transition states corresponding to the low energy barriers. Thus, CEES or DEDS, when entering the micropores, might be “trapped” within the sulfur structures, as shown in Figure 7e–h, and interact favorably not only with the surface (from the bottom) but also with the adsorbed sulfur. The calculated interaction energies of CEES in configurations A and B (Table S2 and Figure 7e,f) are 24.55 and 22.90 kcal/mol, respectively. For DEDS in configurations A and B, these energies are 23.30 and 23.28 kcal/mol, respectively (Figure 7g,h). Different clusters most probably exhibit similar interaction energies. This is because changing the arrangement of sulfur structures around the CEES and DEDS molecules would lead to different “conformers” with similar IE values due to dispersive nature of S_4_–CEES and S_4_–DEDS interactions. These values are much higher than those found for adsorbed CEES and DEDS on pristine graphene (15.81 and 14.19 kcal/mol, respectively) (Figure 8). These results indicate that a small amount of sulfur on the carbon surface enhances adsorption of CEES and DEDS due to the additional dispersive interactions, including S_surface_–S_CEES_ and S_surface_–S_DEDS_ interactions. Indeed, some of the S_surface_–S_CEES_ and S_surface_–S_DEDS_ internuclear distances (shown in Figure 7e–h by red arrows) range from 3.2 to 3.8 Å, which are close to double the sulfur van der Waals radius (being ca. 1.8 Å^42^). Such a closeness of the polarizable sulfur atoms in the (in practice) absence of local dipoles (the electronegativity of sulfur is only very slightly larger than that of carbon) indicates stabilization by London forces.

The presence of a small amount of sulfur also stabilizes the 2CE and HEES molecules on the carbon surface. In the case of 2CE, additional stabilization follows from S_surface_–Cl_2CE_ interactions (Figure S9d,e). The obtained interaction energy does not exceed 17 kcal/mol (Table S2 and Figure 8). In the case of the HEES, surface sulfur interacts with S_HEES_, like in the case of CEES and DEDS (Figure S9f,g), but the interaction energy is lower than those for CEES and DEDS, unless hydrogen bonds between the HEES molecule and the surface functional group is formed, as in the case of graphene–OH (Table S2 and Figure 8).

DFT calculations clearly showed that elemental sulfur adsorbed in the carbon pores in the form of small structures, like S_4_, through dispersive interactions (Figure 7b), which likely exist when 5% of the pore volume is filled, promotes the adsorption of the CEES molecules. This is achieved by both S_surface_–S_CEES_ and S_surface_–Cl_CEES_ dispersive interactions, in addition to interactions with carbon pore walls. The results of DFT calculations also supported the adsorption/desorption behavior discussed above of DEDS, 2CE, and HEES on carbons whose pores are partially filled with sulfur. Thus, DEDS is immobilized on the surface, owing to its energy of interaction with the sulfur-modified surface exceeding 23 kcal/mol, while 2CE could desorb at room temperature due to its interaction energy of less than 20 kcal/mol (Table S2 and Figure 8). With regard to less HEES being extracted from sulfur-containing carbons, the results of the DFT calculations supported the idea that S_surface_–S_HEES_ interactions (more than 20 kcal/mol) could contribute to its retention that might be even enhanced with the strong interactions of HEES with surface polar groups such as OH (close to 30 kcal/mol). With regard to the effects of graphene functional groups on CEES adsorption, the interaction energies in these configurations are quite similar to that on pristine graphene and lower than that on graphene with adsorbed sulfur in both discussed configurations (Figure 8a and Table S2). The changes in the surface composition upon extraction and the extraction efficiencies discussed above strongly agree with the calculated interaction energies, which is reflected in the desorption energies. Nevertheless, one should be aware that other factors, not discussed here, could also contribute to extraction efficiency. They include a strong adsorption potential in confined spaces with regard to adsorbed species and solvent, limited solvent accessibility, and partial pore blocking with sulfur.

## 4 Conclusions

Sulfur tuning of carbon porosity and chemistry markedly affected the interactions of CEES in terms of the strength and extent of its adsorption. A larger surface area and a greater porosity enhanced CEES adsorption by facilitating the uptake of liquid droplets and suppressing the release of toxic vapors. Due to pore blockage caused by the high loading of sulfur in STOC-50, STONC-50, and STUC-50, these samples were inefficient in adsorbing CEES. Differential scanning calorimetry results, measured under CEES pore saturation conditions, further demonstrated that the total heat released upon CEES adsorption was higher for carbon samples without sulfur than for those with sulfur (5% of *V*_T_), stressing the importance of the accessible surface area of the carbons for CEES uptake. However, the results of the thermal analysis and desorption profiles after CEES extraction indicated that sulfur confined within micropores, particularly the ultramicropores of STOC-5, STONC-5, and STUC-5, promoted the retention of CEES molecules, through S_surface_–S_CEES_ dispersive interactions. These findings suggest that while the overall CEES adsorption uptake was largely controlled by the accessible surface area and porosity, the presence of sulfur in ultramicropores decreased the efficiency of CEES extraction. Overall, this study underscores the importance of optimizing the interplay among surface chemistry, porosity, and sulfur loading for the development of efficient materials against chemical warfare agents.

## Supplementary Material



## Notes

Density of dissociating groups, relationship between the chromatographic peak areas of CEES detected in the extract and chromatographic peak areas of CEES detected in the headspace, DTG curves and *m*/*z* thermal profiles for tested carbons, dependence of the chromatographic peak areas of CEES detected in the headspace on the weight loss related to CEES removal, TG and DTG curves of pure CEES measured in argon and thermal profiles of released *m*/*z* fragments, heat flow curves, dependence of the energy of interaction on the surface area, visualization of the interactions of HEES, 2CE, DEDS, and CEES with modified graphene surfaces, and interaction energies (in kilocalories per mole) (PDF)
