# Supplementary material for: Insight into the Role of Sulfur Confined within the Porous Carbon Structure in 2‑Chloroethyl Ethyl Sulfide Adsorption
Source: Langmuir. 2026 Oct 27;42(44):33129–40. doi: 10.1021/acs.langmuir.6c05969 (PMC13757949; doi:10.1021/acs.langmuir.6c05969)
Supplement: Supplementary file 1 [file la6c05969_si_001.pdf]

Supporting Information

Insight into the role of sulfur confined within the porous carbon structure in 2-chloroethyl ethyl sulfide adsorption

Paola S. Pauletto[1], Marc Florent[1], Jingwen Wang[1], Piotr Borowski[2], Teresa J. Bandosz[1*]

[1]Department of Chemistry and Biochemistry, The City College of New York, 160 Convent Ave, New York, NY, 10031, USA

[2]Faculty of Chemistry, Maria Curie-Sklodowska University, Pl. Marii Curie-Sklodowskiej 5, 20-031 Lublin, Poland

[*]Corresponding author: tbandosz@ccny.cuny.edu, (T.J.Bandosz)

Table S1: Density of dissociating groups on mesopore surface of carbons.

Sample	Mesopore surface area (m^2/g)	Total of surface functional groups (mmol/g)	Density of functional groups ($mmol/m^2$)
STOC-0	391	0.56	1.4×10^{-3}
STOC-5	411	0.33	0.8×10^{-3}
STOC-10	395	0.34	0.9×10^{-3}
STOC-50	30	0.04	1.3×10^{-3}
STONC-0	313	1.53	4.9×10^{-3}
STONC-5	320	1.32	4.1×10^{-3}
STONC-10	312	1.20	3.9×10^{-3}
STONC-50	44	0.42	9.6×10^{-3}
STUC-0	386	0.71	1.8×10^{-3}
STUC-5	454	0.67	1.5×10^{-3}
STUC-10	307	0.20	0.7×10^{-3}
STUC-50	30	0.07	2.3×10^{-3}

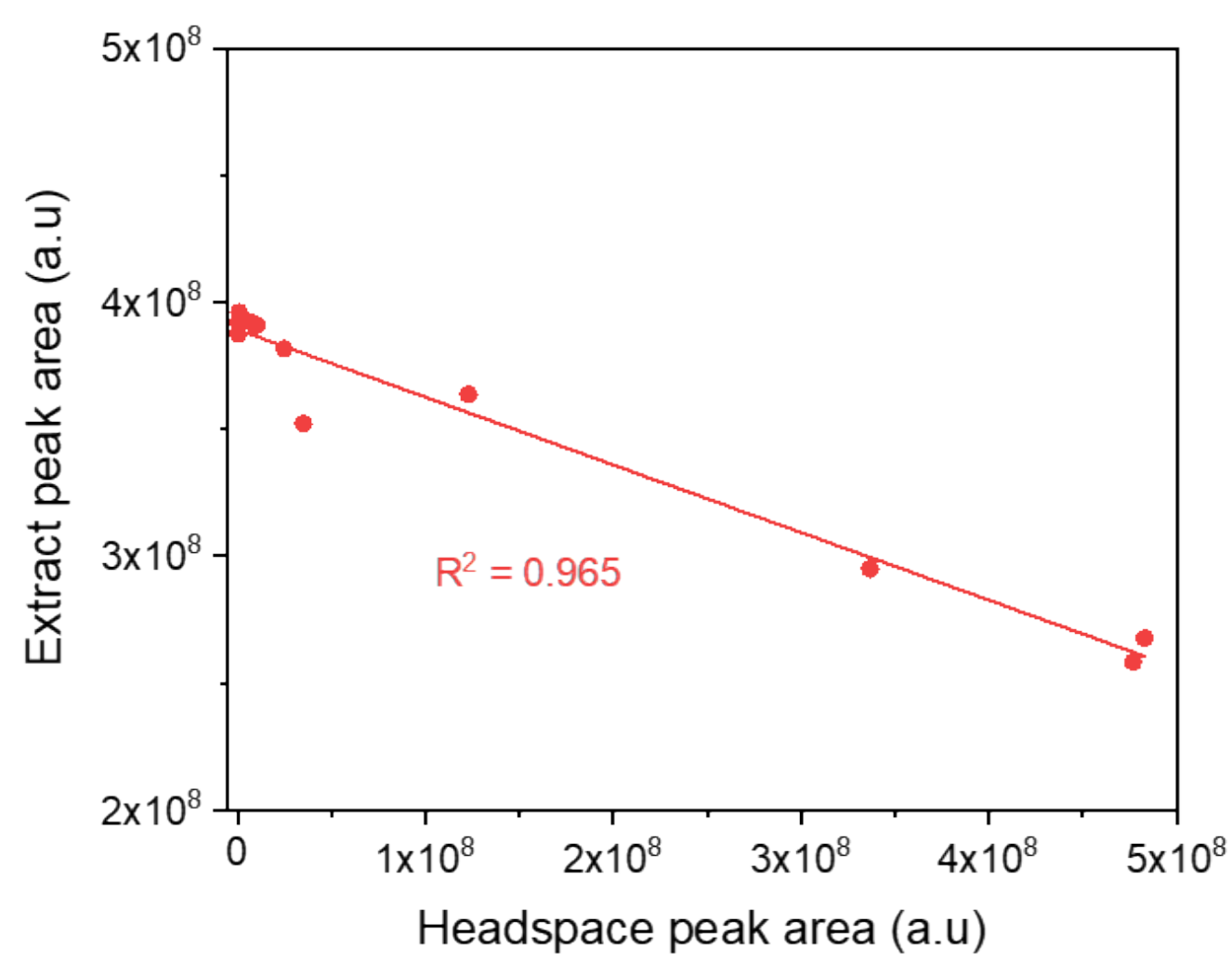

Figure S1: Relationship between the chromatographic peak areas of CEES detected in the extract and chromatographic peak areas of CEES detected in the headspace

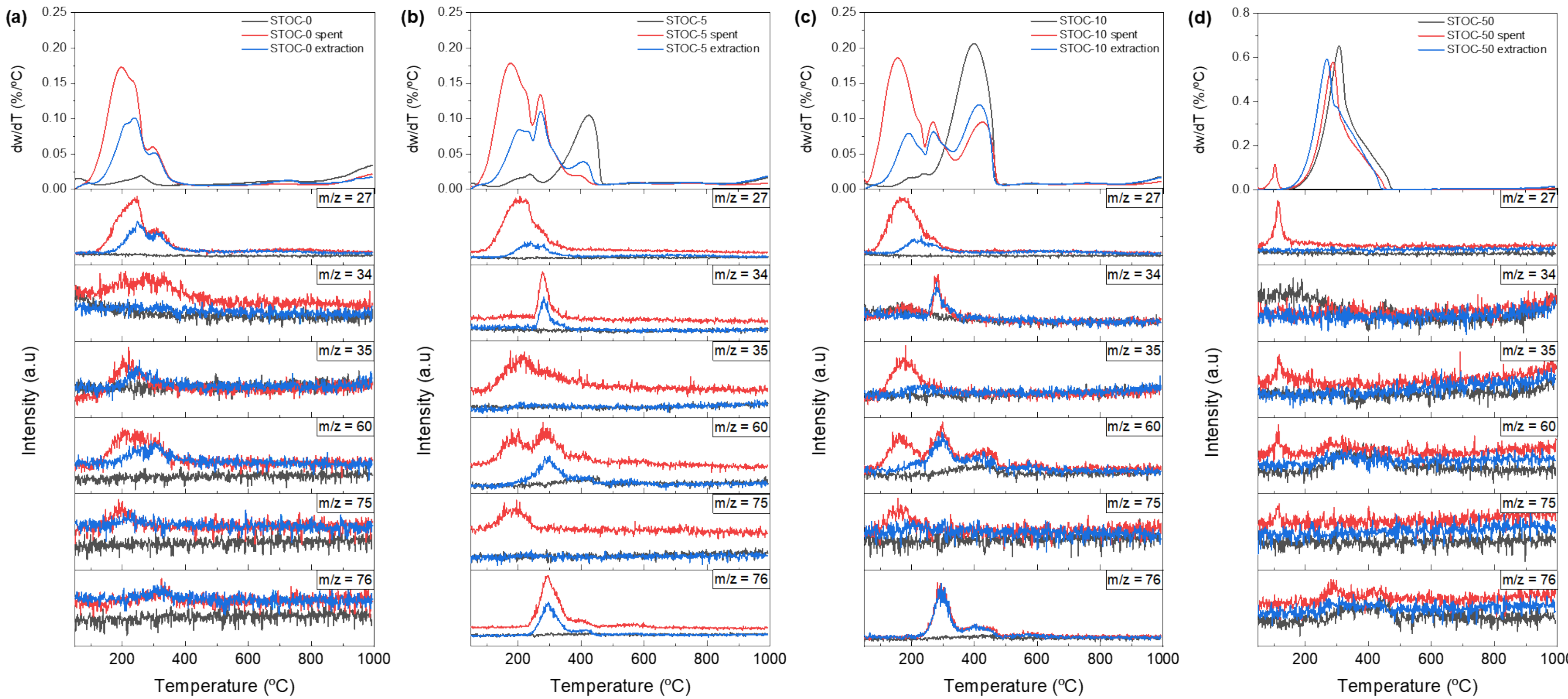

Figure S2: DTG curves and m/z thermal profiles for (a) STOC-0, (b) STOC-5, (c) STOC-10, and (d) STOC-50.

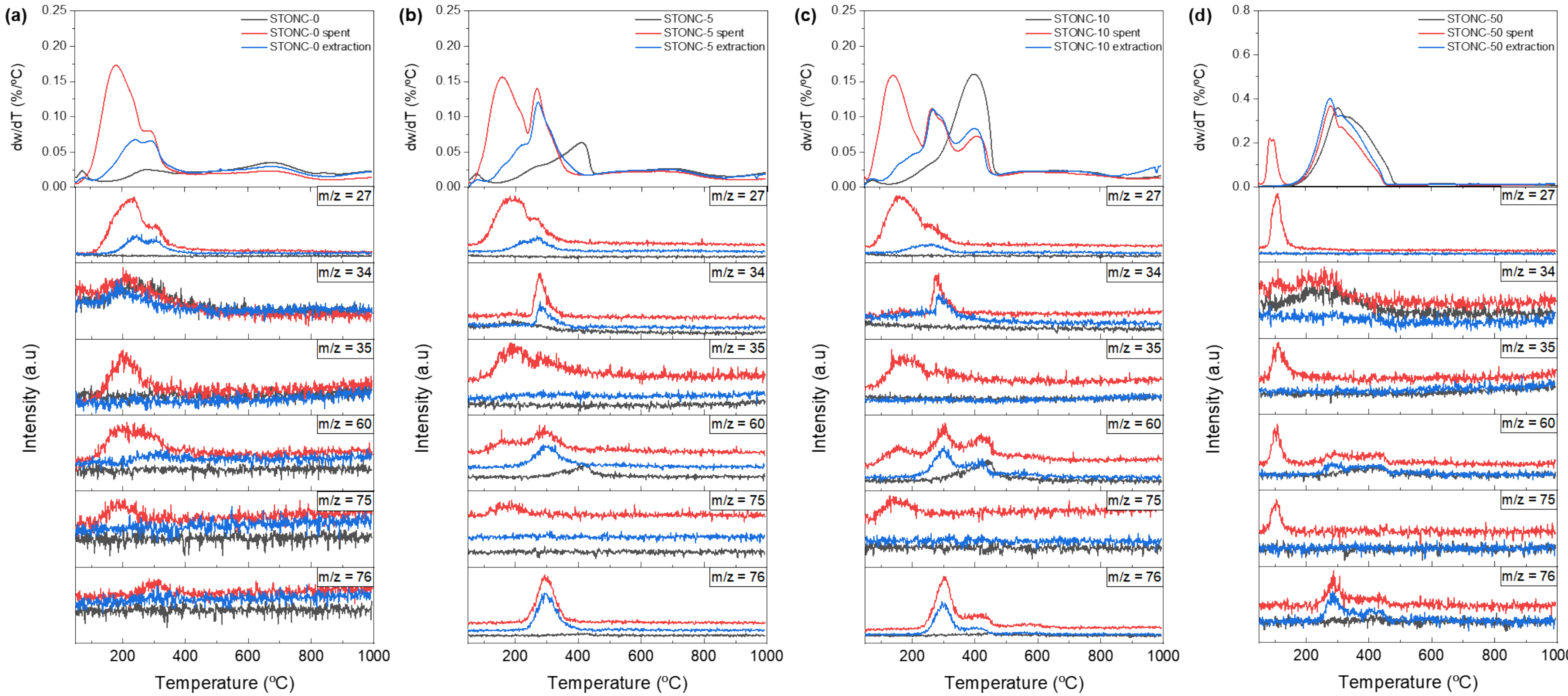

Figure S3: DTG curves and m/z thermal profiles for (a) STONC-0, (b) STONC-5, (c) STONC-10, and (d) STONC-50.

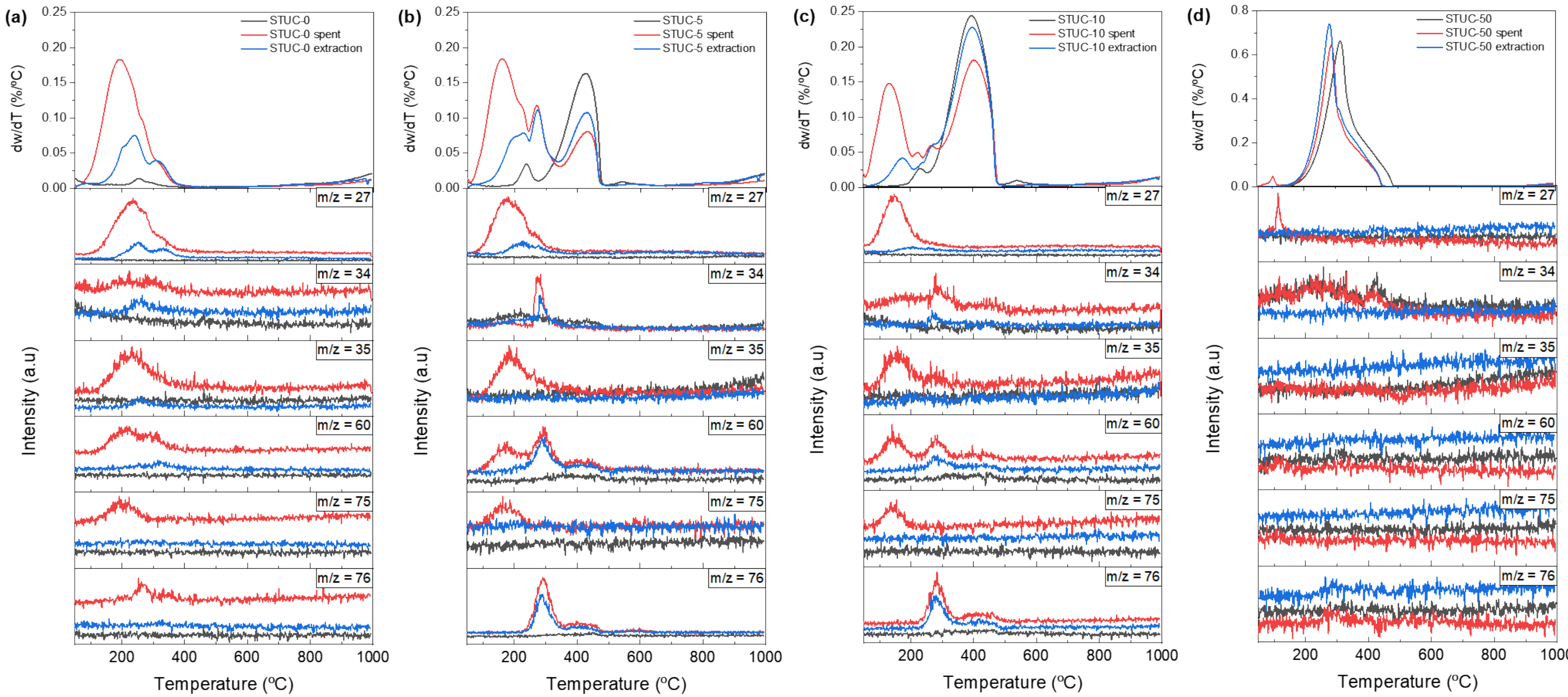

Figure S4: DTG curves and m/z thermal profiles for (a) STUC-0, (b) STUC-5, (c) STUC-10, and (d) STUC-50.

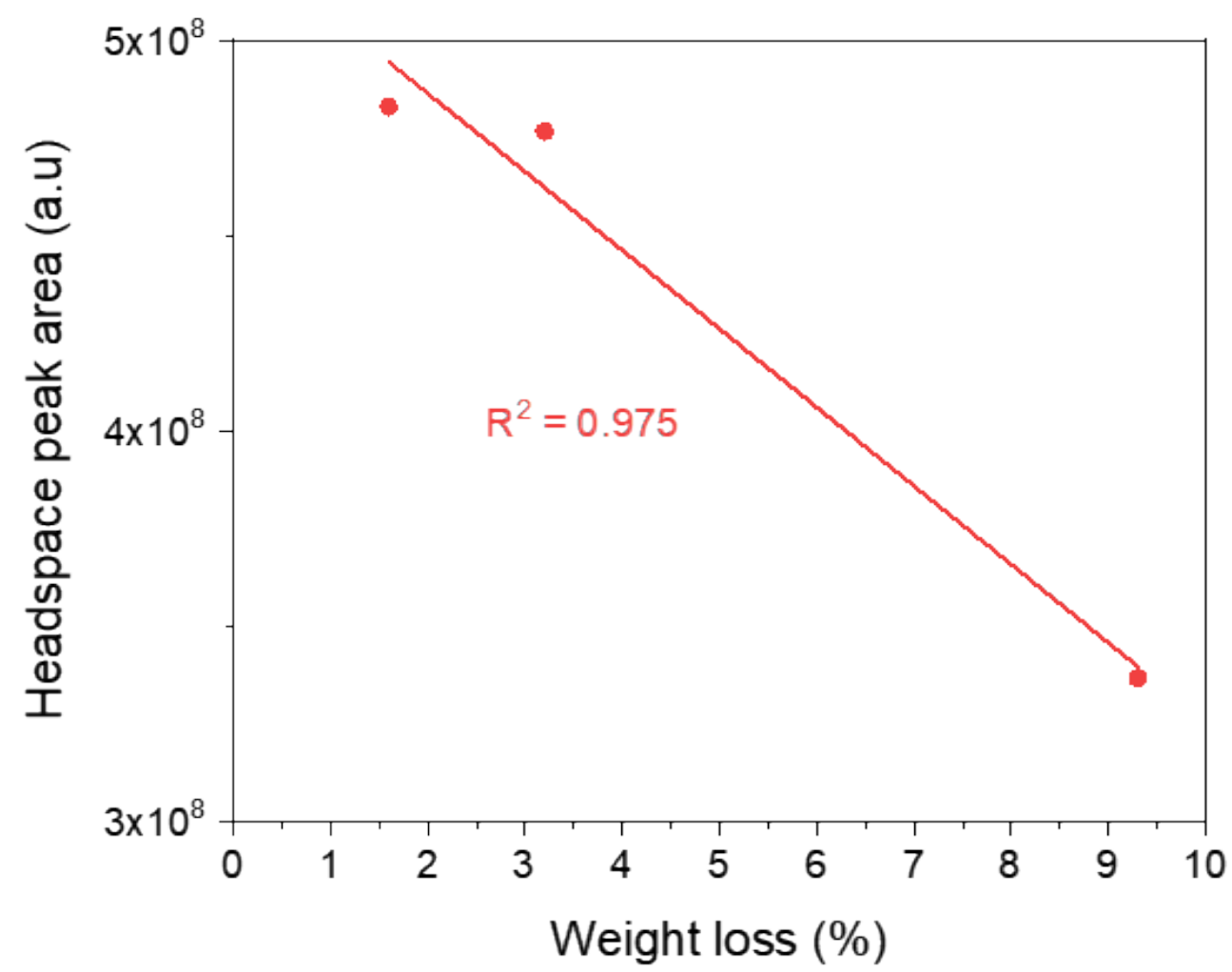

Figure S5: Dependence of the chromatographic peak areas of CEES detected in the headspace on the weight loss related to CEES removal, as determined from the thermal analysis.

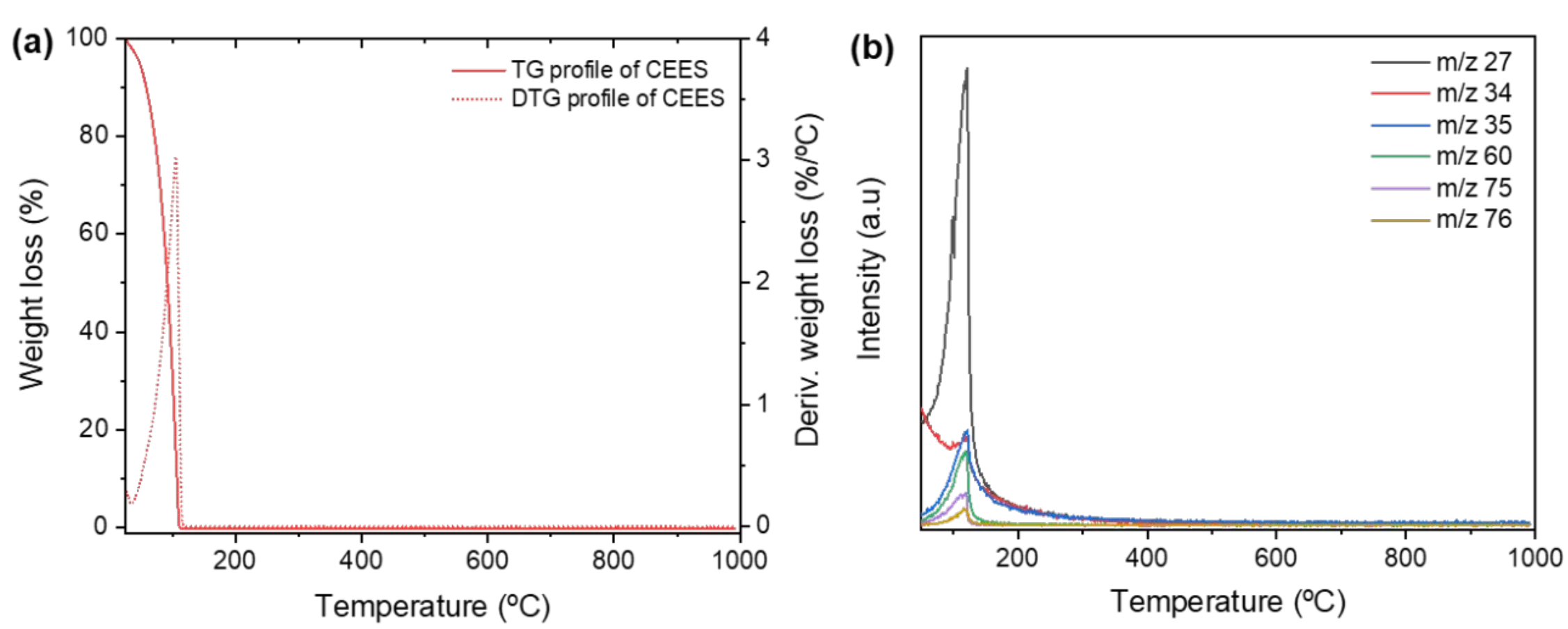

Figure S6: (a) TG and DTG curves of pure CEES measured in argon and (b) thermal profiles of released m/z fragments.

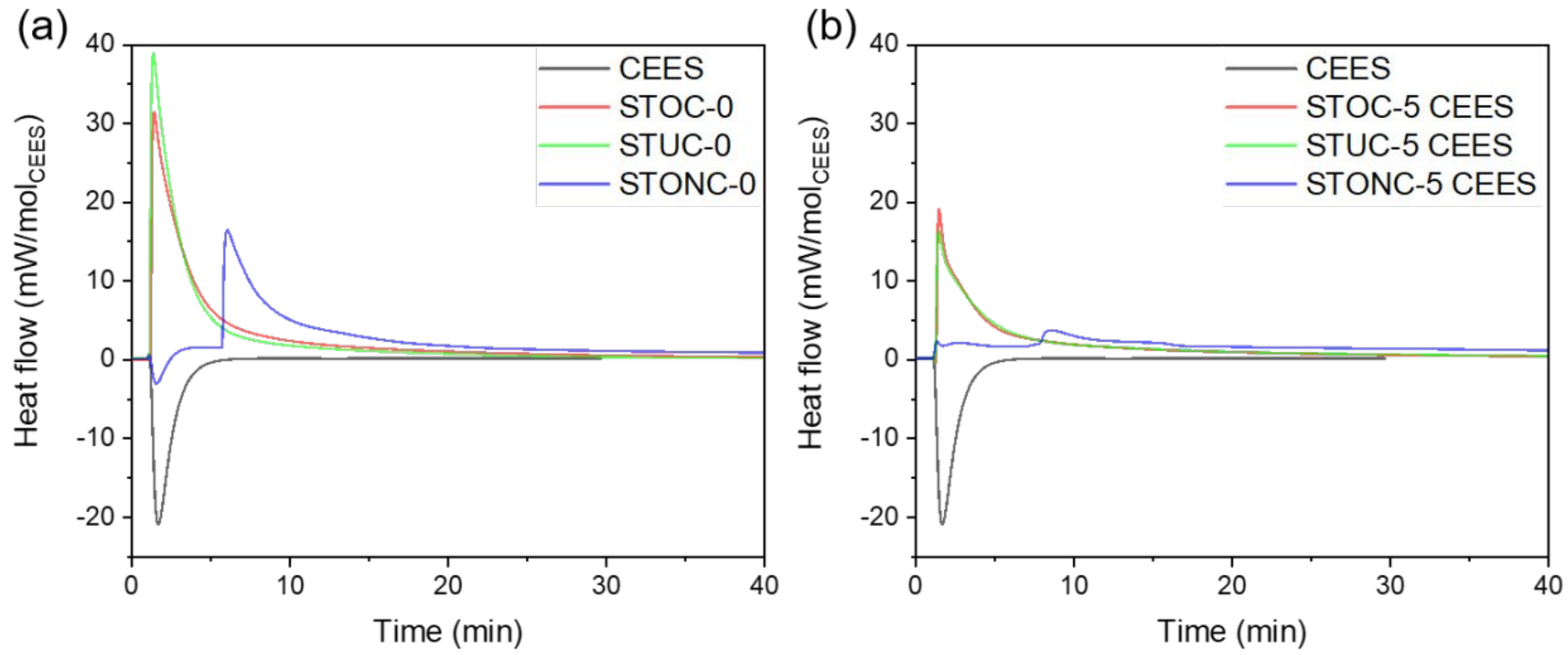

Figure S7: Heat flow curves measured during CEES interaction with carbons (a) without and (b) with 5 % of V_T filled with sulfur.

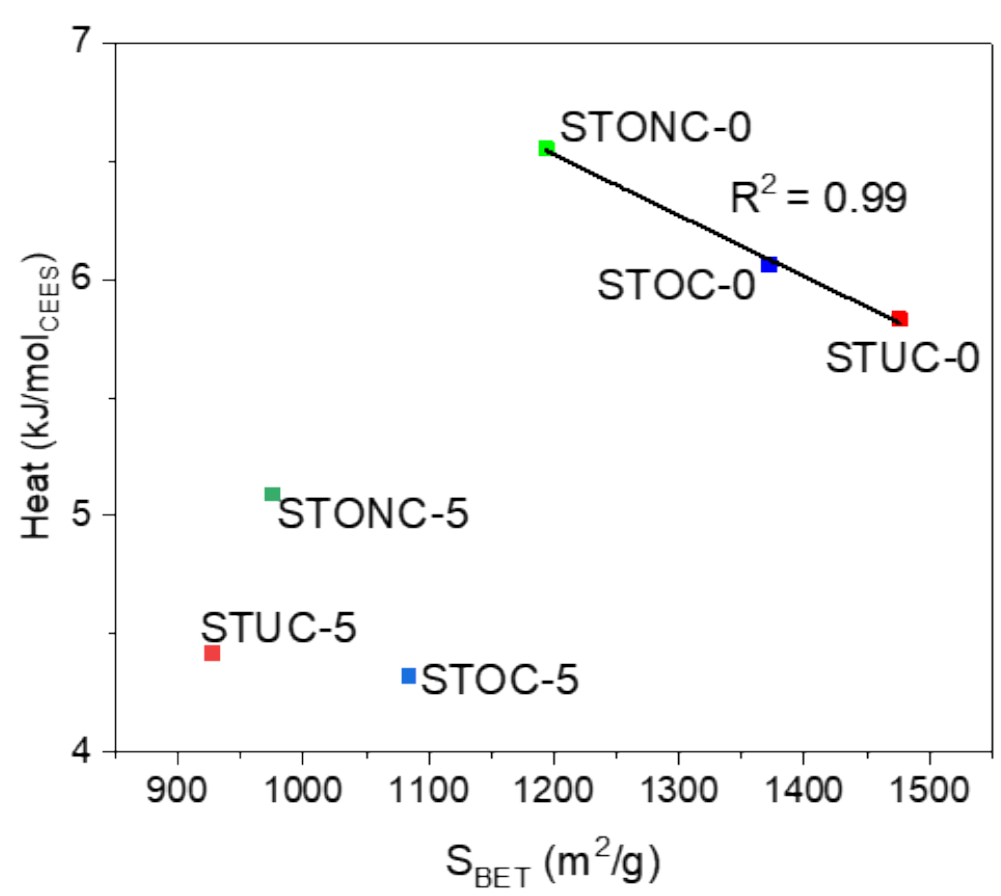

Figure S8: Dependence of the energy of interaction on the surface area of the adsorbents.

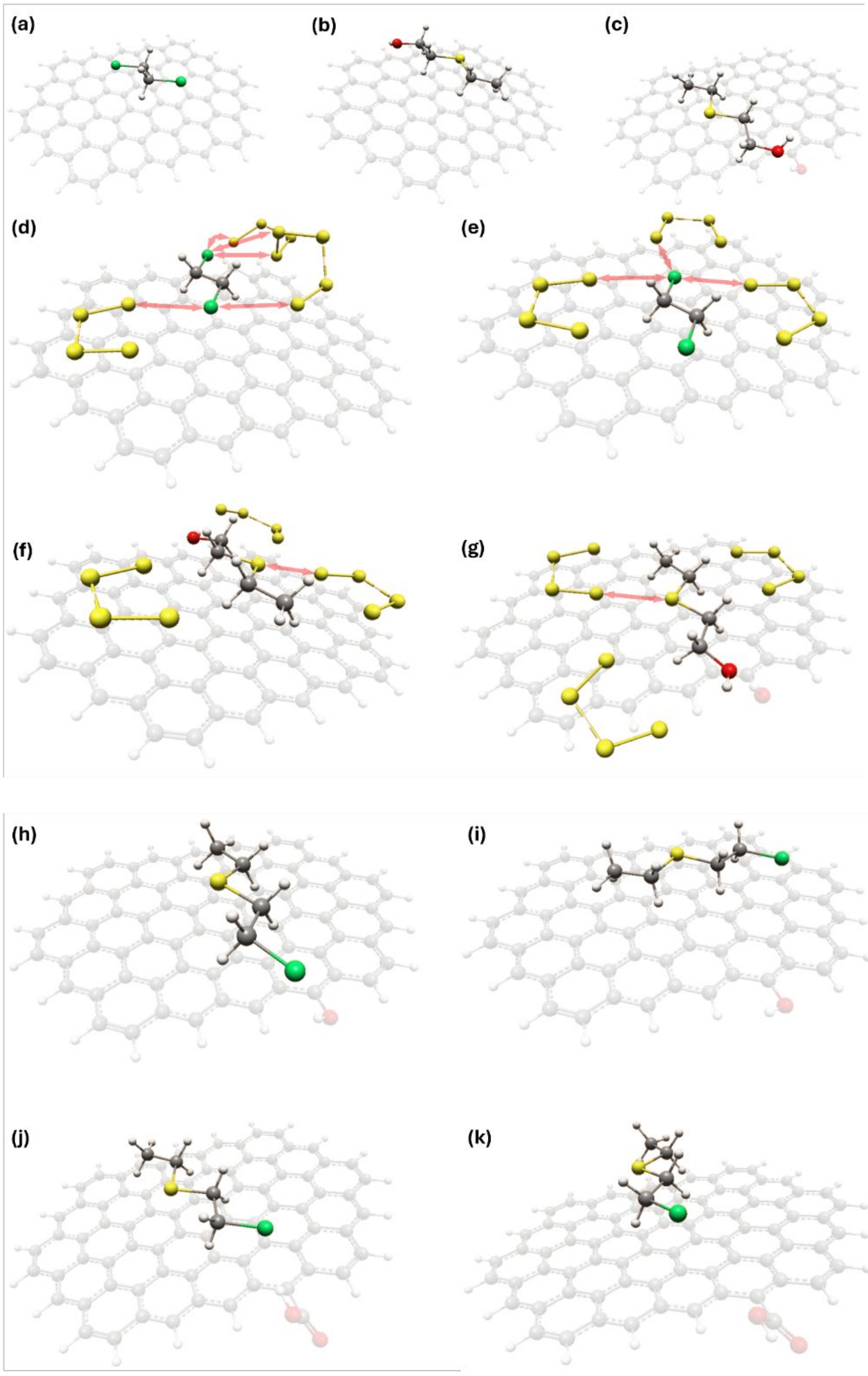

Figure S9. Interactions of HEES with pristine graphene (b), functionalized (OH group) graphene (c) and sulfurized graphene (f, g); interactions of 2CE with pristine graphene (a) and sulfurized graphene (d, e); interactions of CEES with OH (h, i) and COOH (j, k) functionalized graphene. Red arrows indicate $S_{surface}$-Cl_{2CE} and $S_{surface}$-S_{HEES} interactions, in which internuclear distances are in the range from 3.2 Å to 3.8 Å.

Table S2. Interaction energies (IE), basis set superposition errors (BSSE), and BSSE corrected interaction energies (IE+BSSE) (in kcal/mol) for systems considered in this work (shown in Figures 7 and S9). Colors in rows of the table follow those used in Figure 8.

**System**	**IE**	**BSSE**	**IE+BSSE**
Graphene fragment, Fig. 7a	–	–	–
Graphene + S, Fig 7b	58.04[a]	8.88[a]	**49.16[a]**
	67.77[b]	12.41[b]	**55.36[b]**
Graphene + CEES, Fig. 7c	18.05	2.24	**15.81**
Graphene + DEDS, Fig. 7d	16.28	2.09	**14.19**
Graphene + 2CE, Fig. S9a	12.25	1.58	**10.67**
Graphene + HEES, Fig. S9b	18.54	3.51	**15.03**
Graphene-OH + HEES, Fig. S9c	23.52[c]	3.78[c]	**19.74[c]**
Graphene + S + CEES (A), Fig. 7e	31.23	6.68	**24.55**
Graphene + S + CEES (B), Fig. 7f	28.68	5.78	**22.90**
Graphene + S + DEDS (A), Fig. 7g	30.18	6.88	**23.30**
Graphene + S + DEDS (B), Fig. 7h	30.03	6.75	**23.28**
Graphene + S + 2CE (A), Fig. S9d	20.78	3.83	**16.95**
Graphene + S + 2CE (B), Fig. S9e	20.54	4.35	**16.19**
Graphene + S + HEES, Fig. S9f	28.11	7.34	**20.77**
Graphene-OH + S + HEES, Fig. S9g	37.58[c]	7.96[c]	**29.62[c]**
Graphene-OH + CEES (A), Fig. S9h	18.72	2.25	**16.47**
Graphene-OH + CEES (B), Fig. S9i	18.04	2.23	**15.81**
Graphene-COOH + CEES (A), Fig. S9j	20.76	2.56	**18.20**
Graphene-COOH + CEES (B), Fig. S9k	17.27	2.15	**15.12**

[a] Interaction energy of Graphene with $(S_4)_3$ configuration. [b] Interaction energy of Graphene with $3S_4$ configuration. [c] Note that hydrogen bond between surface and adsorbate is formed.
